# Fasting Serum C‐Peptide Levels Predict Cardiovascular and Overall Death in Nondiabetic Adults

**DOI:** 10.1161/JAHA.112.003152

**Published:** 2012-12-19

**Authors:** Nileshkumar Patel, Tracey H. Taveira, Gaurav Choudhary, Hilary Whitlatch, Wen‐Chih Wu

**Affiliations:** 1 Research Enhancement Award Program, Providence Veterans Affairs Medical Center, Providence, RI (N.P., T.H.T., W.C.W.); 2 University of Rhode Island College of Pharmacy, Kingston, RI (T.H.T., W.C.W.); 3 Medical Service, Providence Veterans Affairs Medical Center, Providence, RI (G.C., H.W., W.C.W.); 4 Pharmacy Service, Providence Veterans Affairs Medical Center, Providence, RI (T.H.T.); 5 Department of Medicine, Alpert Medical School of Brown University, Providence, RI (T.H.T., G.C., H.W., W.C.W.)

**Keywords:** cardiovascular risk, glucose intolerance, insulin resistance

## Abstract

**Background:**

Insulin resistance, characterized by hyperinsulinemia and normal or elevated serum glucose, is an established precursor to diabetes and cardiovascular disease. Despite fasting serum C‐peptide levels being an accurate and stable marker of endogenous insulin production used in patients with diabetes, it is unknown whether C‐peptide could serve as a marker of insulin resistance and predict outcomes in patients without diabetes.

**Method and Results:**

This is a retrospective cohort study using data from the NHANES‐3 (1988–1994) survey with mortality follow‐up through December 31, 2006. Participants included 5153 subjects, 40 to 74 years of age with fasting glucose ≥70 mg/dL, without diabetes by history or laboratory testing. Receiver‐operating‐curve analysis compared fasting C‐peptide against known insulin resistance measures such as fasting plasma glucose, serum insulin, HOMA‐IR, quantitative‐insulin‐sensitivity‐check‐index, and metabolic syndrome for the prediction of cardiovascular and overall death. Subjects were then stratified by quartiles of C‐peptide levels. Cox proportional‐hazards modeling compared hazards of cardiovascular and overall death amongst C‐peptide quartiles and adjusted for potential confounders of cardiovascular and diabetes risk. Fasting serum C‐peptide levels predicted cardiovascular and overall death better than other studied measures (AUC=0.62 and 0.60 respectively vs the rest, with AUC≤0.58 and ≤0.57 respectively, *P*<0.001). When compared with the lowest C‐peptide quartile, subjects in the highest quartile had significantly higher adjusted hazard ratios (HR) of cardiovascular death (HR=1.60, 95%CI 1.07 to 2.39) and overall mortality (HR=1.72, 95%CI 1.34 to 2.21) after controlling for confounders.

**Conclusions:**

C‐peptide levels significantly related to hazards of cardiovascular and overall death in nondiabetic adults and was a better predictor of these outcomes than serum insulin and/or glucose derived measures.

## Introduction

Insulin resistance is a component of type 2 diabetes^1^ and is associated with obesity^2^ and glucose, lipid and blood pressure dysregulation,^3^ in a conglomeration of cardiovascular risk factors defined as metabolic syndrome.^4–5^ Insulin resistance is characterized by hyperinsulinemia^6^ in the setting of normal or elevated serum glucose and has been related to incident diabetes,^7^ coronary heart disease,^8–9^ and mortality^10–11^ in subgroups of nondiabetic subjects. Therefore, assessment of insulin resistance is an important way to identify individuals without diabetes who are at an increased risk for adverse outcomes and potentially targets them for early intervention.

Previous studies have included measurement of serum insulin levels as a marker of insulin resistance.^7^ An alternative marker is C‐peptide, a protein that is cosecreted with insulin on an equimolar basis from pancreatic beta cells. Unlike insulin, it does not undergo hepatic first pass metabolism, has a longer half‐life, and has been recognized as a more stable and accurate marker of endogenous insulin secretion.^12–14^ However, there is limited data on the clinical and epidemiological value of C‐peptide measurement as a marker of insulin resistance, especially in individuals without known diabetes mellitus. Furthermore, it is unclear how C‐peptide compares to other known indices of insulin resistance in predicting cardiovascular and overall death.

We hypothesized that fasting serum C‐peptide level is a better marker of insulin resistance than insulin level alone and would be useful in the prediction of death related to cardiovascular disease in adults without diabetes. To test this hypothesis, we compared fasting serum C‐peptide level to other measures of insulin resistance as a predictor of future cardiovascular and overall death using biochemical and outcome data from a nationally representative sample of nondiabetic adults living in the United States. Additionally, we studied the relationship between C‐peptide levels and mortality related to cardiovascular diseases, independent of other known cardiovascular or diabetes risk factors.

## Methods

### Design

We conducted a retrospective cohort study to assess the relationship between insulin resistance as measured by fasting C‐peptide levels and subsequent cardiovascular disease and overall death in nondiabetic adults.

### Data Source

The Third National Health and Nutrition Examination Survey (NHANES 3) was a nationwide survey conducted by the Centers for Disease Control and Prevention to assess the health and nutrition status of adults and children in the United States in 1988–1994 through interviews, physical examination, and laboratory assessments.^15^ Because this study involved the use of deidentified data in a publically accessible government database, we obtained an exemption status from the Providence VA Medical Center's institutional review board.

### Study Sample

We studied adults without diabetes, 40 to 74 years of age, who had undergone an oral glucose tolerance test from the NHANES 3 data set (N=6652). Excluded were participants who were told they had diabetes by their physicians or were taking antidiabetes medications (510 excluded) and those who had a fasting plasma glucose ≥126 or <70 mg/dL, an oral glucose tolerance test glucose ≥200 mg/dL, or serum hemoglobin A1c levels of ≥6.5% (989 excluded). This yielded a final analytic sample of 5153 subjects.

### Outcomes

The primary outcome was cardiovascular death, and the secondary outcome was overall (all‐cause) death. Tertiary outcomes included subcategories of the primary outcome such as cerebrovascular disease death, ischemic heart disease death, and myocardial infarction death. Mortality status was obtained from the NHANES 3–linked mortality‐public‐use file, which provided mortality data from the date of the NHANES 3 survey participation through December 31, 2006.^15^ Underlying causes of death were provided by death certificate data contained in the same mortality files and classified according to the *10th International Statistical Classification of Diseases, Injuries and Causes of Death* (ICD‐10) guidelines. Cardiovascular death was considered when cardiovascular disease was the underlying cause of death or when it is one of the multiple causes of the death. The ICD‐10 codes for cardiovascular disease (I00 to I99) were identified according to the American Heart Association guidelines and included essential hypertension and hypertensive heart and kidney disease (I10 to I13), ischemic heart diseases (I20 to I25), acute rheumatic fever and chronic rheumatic heart diseases (I00 to I09), acute and subacute endocarditis (I33), diseases of pericardium and acute myocarditis (I30 to I31, I40), other heart diseases (I26 to I51), heart failure (I50), all other forms of heart disease (I26 to I28, I34 to I38, I42 to I49, I51), cerebrovascular diseases (I60 to I69), atherosclerosis (I70), and other diseases and disorders of circulatory system (I71 to I78, I80 to I99).^16^ The distribution of major cardiovascular diseases in death certificates of the study population was included in Table 1. Follow‐up data were 99.8% complete, with the exception of 1 participant without survival data and 12 participants whose underlying cause of death cannot be ascertained because death certificate data were not available.

**Table 1.** Distribution of Major Cardiovascular Diseases in Death Certificates of the Study Population

Ischemic heart diseases, %	50.6
Cerebrovascular diseases, %	14.84
Hypertension as multiple causes of death, %	8.74
Hypertensive heart disease, %	2.64
Hypertensive heart and renal disease, %	0.41
Essential (primary) hypertension and hypertensive renal disease, %	1.83
Heart failure, %	5.89
Aortic aneurysm and dissection, %	2.24
Other diseases of arteries, arterioles and capillaries, %	0.20
Other disorders of circulatory system, %	0.81
Atherosclerosis, %	0.81
All other forms of heart disease*, %	10.37
Acute rheumatic fever and chronic rheumatic heart diseases, %	0.41
Acute and subacute endocarditis, %	0.20

* Pulmonary heart disease and diseases of pulmonary circulation (eg, pulmonary embolism), valvular heart disease, cardiomyopathy, arrhythmia (tachy and brady), cardiac septal disease, rupture of chordae tendinae, myocarditis, myocardial degeneration, and Takotsubo syndrome.

Fasting serum C‐peptide levels were drawn in conjunction with baseline plasma glucose as part of the participant's oral glucose tolerance test and excluded participants with any of the following conditions: diagnosis of diabetes mellitus, prescribed medications for diabetes, diagnosis of hemophilia, cancer with chemotherapy 4 weeks before the examination, or pregnancy. The eligible participants were instructed to fast for 10 to 16 hours before the morning blood draw. The specimen was obtained at the time of the initial venipuncture and processed using standard radioimmunoassay methods by highly trained medical personnel who had completed comprehensive training in standardized laboratory procedures. Equipment operation, specimen collection and preparation, and testing procedures were monitored for quality assurance with detailed instructions in the manual of medical technicians (US Department of Health and Human Services, 1996).^17–18^ The minimal reportable range for C‐peptide levels was 0.021 nmol/L; values below the detection limit were coded in this study as 0.021. The coefficient of variation for the lower extreme of the values was 14.7% (for mean C‐peptide levels of 0.265, 95% confidence interval [CI] 0.187 to 0.343 nmol/L) and 6.6% for the upper extreme of the values (for mean C‐peptide levels of 3.311, 95% CI 2.875 to 3.747 nmol/L).^18^

Covariates that were studied at baseline for potential confounding assessment included standard demographics such as age, sex, ethnicity (white, black, Mexican, and other) and education level (less than, equivalent to, and above high school). Based on previous literature, we also assessed the following variables that are known to relate to the development of diabetes or cardiovascular disease: alcohol use (>1 for female and >2 for male, ≤1 for female and ≤2 for male, and 0, drinks in the last month),^19^ physical activity (none, <3, and ≥3, times per week),^20^ fasting plasma glucose levels,^21^ glycated hemoglobin (A1c) levels,^22^ body mass index,^20^ estimated glomerular filtration rate by Modification of Diet in Renal Disease (MDRD) formula^23^ (≥90, 60 to 89, and <60 mL/min),^24^ urinary albumin/creatinine ratio,^25^ and C‐reactive protein levels.^26–27^ Also included were cardiovascular risk factors from the Third Adult Treatment Panel (ATP 3) guidelines^5^ such as smoking status (current, former, and never), blood pressure (<130/80, 130 to 139 systolic or 80 to 89 diastolic, and ≥140 systolic or 90 diastolic, mm Hg), total cholesterol, high‐density lipoprotein (HDL) cholesterol, and calculated low‐density lipoprotein (LDL) cholesterol (total cholesterol−[triglycerides/5]−HDL)^28^; components of metabolic syndrome not already included from the same guidelines (triglycerides, waist‐to‐hip ratio),^5^ family history of heart attack or diabetes, as well as a personal history of stroke, heart attack, chest pain (none, suggestive, and not suggestive, of angina), and peripheral arterial disease.

### Statistical Analysis

Given the complex nonrandom multistage stratified sample design of NHANES 3, analyses were performed using the designated weighting specified in the data set to minimize biases.^29^ As such, we used the total NHANES 3 pseudostratum as our strata variable, the total pseudoprimary sampling units as our survey sampling units, and the total mobile examination center (MEC) final weight as our sampling unit weight.

First, we evaluated the sensitivity and specificity of several indices of insulin resistance in the prediction of cardiovascular and overall death by means of receiver operating characteristic (ROC) curves and evaluated their correlation with C‐peptide levels. The indices included fasting levels of serum C‐peptide, serum insulin,^6^ and plasma glucose, as well as the homeostatic model assessment of insulin resistance (HOMA‐IR index),^30^ quantitative insulin sensitivity check index (QUICKI index),^31^ and metabolic syndrome.^5^ Next, we identified the index of insulin resistance with the largest ROC area under the curve (AUC) and used it as the reference for comparison against the AUC of other indices using chi‐square analysis. The ROC analyses were frequency weighted using NHANES 3 probability weights rounded to the nearest integer.^32^

We then stratified our study sample by quartiles of C‐peptide levels (≤0.418, 0.419 to 0.652, 0.653 to 0.983, and ≥0.984 nmol/L). Differences in baseline characteristics among subjects across C‐peptide quartiles were compared using linear regression for continuous variables and χ^2^ analysis for categorical variables. Kaplan–Meier curves were created to estimate the percent of event‐free period of survival according to C‐peptide quartiles. The association between C‐peptide quartiles and study outcomes over time was examined using Cox proportional‐hazards modeling with subjects in the lowest C‐peptide quartile (≤0.418 nmol/L) as the reference category. We adjusted for potential confounding in a model that included all the assessed covariates with the exception of LDL cholesterol due to its high collinearity with other lipid variables. The resulting model yielded excellent discrimination with a Harrell's C statistic of 0.82 for cardiovascular mortality and 0.78 for overall mortality. We tested for assumption of proportional hazards on the primary outcome by visual inspection of the Kaplan–Meier curves as well as the inclusion of an interaction term of C‐peptide quartiles with log(time) in the Cox model.^33^ The comparison of the Cox models with and without the interaction term showed no significant difference by likelihood‐ratio test (*P*=0.94), confirming the assumption of the proportional hazards. We also estimated the attributable risk percent for the cardiovascular and overall death in relationship to C‐peptide levels to determine the excess risk associated with high C‐peptide levels,^34^ where the “exposed” group was defined by the top quartile of C‐peptide levels and the “unexposed” group by the remaining population.

Additional analyses on the primary outcome were performed to test whether potential interaction between C‐peptide levels and body mass index, sex, race, or glomerular filtration rate (≥60 mL/min vs <60 mL/min) existed. In addition, subgroup analyses were performed for individuals with normal fasting glucose (<100 mg/dL) and in those without metabolic syndrome as defined by the ATP 3 criteria,^5^ and in specific outcomes of interest; namely, death due to cerebrovascular disease, ischemic heart disease, and myocardial infarction (included in the ischemic heart disease death). To identify whether the association with mortality differed for C‐peptide compared with other biochemical markers of insulin resistance (HOMA‐IR, QUICKI, and serum insulin levels), we also studied the effect of using these other biochemical markers in lieu of C‐peptide quartiles in the survival models.

Data was 98.6% complete for all covariates, with missing values that ranged from 0.1% (history of peripheral arterial disease) to 2.5% (waist‐to‐hip ratio). Missing data in the covariates were imputed based on 10 sets of simulated values generated from nonmissing variables using the multiple imputation method in STATA (StataCorp, College Station, Tex).^35^ Analyses were performed on each of the 10 data sets completed with imputed values, and then combined using Rubin's combination rules to consolidate the individual estimates into a single set of estimates using the MI estimate command in STATA.^36–37^ All statistical analyses were conducted in STATA SE 11.1.^38^ A 2‐sided *P* value of <0.05 was considered significant.

## Results

### Baseline Characteristics of the Study Population

Our final analytic sample included 5153 nondiabetic adults between 40 and 74 years of age, which was representative of a population of 61 651 227 nondiabetic US adults of the same age group. The baseline clinical characteristics of the study population, both overall and according to C‐peptide category, are shown in Table 2. Increasing quartiles of C‐peptide levels were associated with higher likelihoods of having an older age, male gender, nonwhite ethnicity, education less than high school, previous or current smoking history, personal history of heart attack, chest pain or stroke; family history of diabetes, blood pressure ≥140/90, and metabolic syndrome. Compared with participants in the lowest C‐peptide quartile, participants in the higher C‐peptide quartiles were also more likely to have a higher waist‐to‐hip ratio and body mass index. Participants in the lowest C‐peptide quartile, on the other hand, were more likely to have exercised ≥3 times a week, to have consumed alcohol in the month before the survey, and to have a blood pressure <130/80 compared with participants in higher C‐peptide quartiles (Table 2).

**Table 2.** Baseline Clinical Characteristics of the Study Population, According to Fasting C‐Peptide Levels

C‐Peptide Category, nmol/L						
	Overall	≤0.418	0.419 to 0.652	0.653 to 0.983	≥0.984	*P* Value
No. of participants	5153	1288	1288	1290	1287	<0.01
Mean age±SD, y	54.9±10.5	53.4±10.2	55.0±10.5	55.1±10.4	56.1±10.7	
Sex, %						<0.01
Male	48.3	42.1	46.1	50.7	56.2	
Female	51.7	57.9	53.9	49.3	43.8	
Ethnicity, %						0.01
White	81.7	83.3	80.8	82.6	80	
Black	8.4	8.7	8.7	8.2	7.7	
Mexican	3.2	2.1	2.8	3.7	4.6	
Others	6.7	5.9	7.7	5.5	7.7	
Education, %						<0.01
Less than high school	23.6	18.9	23.2	25.7	27.5	
High school or equivalent	33.5	32.5	30.7	35.5	36	
College or above	42.4	48.1	45.6	38.3	36.1	
Smoking status, %*						<0.01
Current	25.7	25.6	27.3	24.5	25.1	
Former	33.7	31.2	28.6	36.2	40.3	
Never	40.6	43.2	44.1	39.3	34.6	
History of stroke, %	1.7	1.2	1.4	2.1	2.1	0.58
History of heart attack, %	4.0	2.4	2.7	4.3	7.1	<0.01
History of chest pain, %						<0.01
Suggestive of angina	4.7	3.3	3.8	5.3	7.0	
Not suggestive of angina	28.7	26.6	25.1	30.7	33.2	
No chest pain	66.6	70.1	71.1	64	59.8	
Peripheral arterial disease, %	0.9	0.2	1.3	0.3	2.3	<0.01
Family history of heart attack, %	10.7	10.1	10.9	11.4	10.6	0.51
Family history of diabetes, %	25.3	22.1	24.3	24.9	31.1	0.02
Blood pressure, %						<0.01
<130/80, mm Hg	51.3	66.0	53.9	46.2	35.3	
130 to 139/80 to 89, mm Hg	26.1	19.2	27.4	30.4	28.6	
≥140/90, mm Hg	22.5	14.8	18.4	23.3	36.0	
Alcohol use, %						<0.01
0 drink/last month	46.1	41.2	42.8	48.9	53.2	
≤2 drinks/day if male and ≤1 drink/day if female	49.5	52.8	52.7	47.6	43.4	
>2 drinks/day if male and >1 drink/day if female	4.3	5.8	4.4	3.4	3.3	
Physical activity, %						<0.01
No activity	13.8	11.4	11.3	15.7	17.4	
<3 times/week	31.1	23.6	33.7	33.9	34.5	
Mean waist‐to‐hip ratio±SD	0.9±0.1	0.9±0.1	0.9±0.1	1.0±0.1	1.0±0.1	<0.01
Mean body mass index±SD, kg/m^2^	27.5±5.5	23.9±3.7	26.3±4.3	28.6±4.9	31.2±5.9	<0.01
Metabolic syndrome, %*	24.5	3.4	12.1	32.3	57.4	<0.01

Frequencies (%) in the columns may not sum to 100% to account for missing data.

* Participants were classified into current smoker if he or she had smoked at least 100 cigarettes during his or her entire life and still smokes, former smoker if he or she had smoked at least 100 cigarettes during his or her entire life but not currently smoking; and never if he or she had never smoked >100 cigarettes during his or her entire life.

* Metabolic syndrome is diagnosed when ≥3 of the following criteria are present; waist circumference >102 cm for men and >88 cm for women, triglycerides level >150 mg/dL, high‐density lipoprotein level <40 mg/dL for men and <50 mg/dL for women, blood pressure ≥130/≥85 mm Hg, and fasting glucose ≥110 mg/dL. (Ref Third report of the National Cholesterol Education Program expert panel on detection, evaluation, and treatment of high blood cholesterol in adults)

The baseline biochemical characteristics of the study population, both overall and according to C‐peptide category, are shown in Table 3. Compared with participants in the lowest C‐peptide quartile, participants in the higher C‐peptide quartiles were more likely to have higher plasma glucose during fasting and 2‐hour post‐glucose tolerance test, urinary albumin/creatinine ratio, A1c, total and LDL cholesterol, triglyceride, serum fasting insulin, and C‐reactive protein levels, as well as the HOMA‐IR index. On the other hand, participants in the lowest C‐peptide quartile were more likely to have a higher HDL cholesterol level, QUICKI index, and glomerular filtration rate ≥90 mL/min than were participants in the higher C‐peptide quartiles.

**Table 3.** Baseline Biochemical Characteristics of the Study Population, According to Fasting C‐Peptide Levels

C‐Peptide Category, nmol/L						
	Overall	≤0.418	0.419 to 0.652	0.653 to 0.983	≥0.984	*P* Value
No. of participants	5153	1288	1288	1290	1287	<0.01
Mean hemoglobin A1c±SD, %	5.4±0.5	5.3±0.4	5.4±0.4	5.4±0.5	5.5±0.5	
Glomerular filtration rate, %*						<0.01
≥90, mL/min	4.3	4.6	4.2	4.8	3.5	
60 to 89, mL/min	70.3	76.7	71.7	69.8	61.2	
<60, mL/min	24.2	17.4	23.3	23.8	34.2	
Mean fasting plasma glucose±SD, mg/dL	95.5±9.2	90.9±7.5	94.8±8.2	96.8±8.8	99.5±9.8	<0.01
Mean 2 h OGTT±SD, mg/dL	121.2±34	115.6±34.8	117.2±32.9	124.4±33.4	127.2±33.3	<0.01
Mean serum cholesterol±SD, mg/dL	220.6±43.1	213.9±41.9	221.3±40.4	222.2±44.8	224.9±44.3	<0.01
Mean serum HDL±SD, mg/dL	51.5±16.2	59.6±17.5	53.4±15.9	48.4±13.9	44.7±13.2	<0.01
Mean serum triglycerides±SD, mg/dL	149.4±106.2	105.6±69.7	128.8±76.8	161.4±106.2	201.9±133.8	<0.01
Mean calculated serum LDL±D, mg/dL*	140.4±38.7	133.9±38.3	142.8±37.7	142.5±39.5	142.6±38.8	<0.01
Mean serum insulin±SD, pmol/L	65.2±47.3	34.4±12.7	47.6±13.4	64.7±19.1	114.2±67.8	<0.01
Mean serum CRP±SD, mg/dL*	0.5±0.7	0.4±0.6	0.4±0.7	0.5±0.8	0.6±0.7	<0.01
Mean urinary albumin/creatinineratio±SD, mg/g	21.2±110.9	10.3±34.8	16.5±94.2	23.3±127.3	34.8±150.6	<0.01
Mean HOMA*	15.6±12.5	7.8±3.0	11.2±3.4	15.5±4.8	28.2±18.5	<0.01
Mean QUICKI*	0.27±0.02	0.29±0.01	0.28±0.01	0.27±0.01	0.25±0.01	<0.01

Frequencies (%) in the columns may not sum to 100% to account for missing data.

OGTT indicates oral glucose tolerance test; HDL, high‐density lipoprotein; LDL, low‐density lipoprotein; CRP, C‐reactive protein; HOMA, homeostatic model assessment; QUICKI, quantitative insulin sensitivity check index.

* MDRD formula was used to calculate GFR. GFR=186*serum creatinine−1.154·age−0.203·1.210 (for black)·0.742 (for female). GFR is in mL/min/1.73 m^2^ body surface area.

* LDL was calculated using following formula: LDL=total cholesterol−HDL−0.20·serum triglycerides if triglycerides ≤400 mg/dL. For triglycerides >400 mg/dL, LDL value was considered missing.

* Lowest detection limit for biochemistry test used in NHANES 3 for C‐reactive protein was 0.3 mg/dL.

* HOMA=fasting plasma glucose (mg/dL)×fasting serum insulin levels/405.

* QUICK index=1/(log [fasting serum insulin]+log [fasting plasma glucose]).

### Comparison of C‐Peptide With Other Indices of Insulin Resistance

C‐peptide levels had a high correlation (*P*<0.001) with other biochemical indices of insulin resistance such as HOMA‐IR (*r*=0.78), QUICKI (*r*=−0.78), and serum insulin levels (*r*=0.77). ROC curve analyses showed that fasting serum C‐peptide levels had the largest AUC in the prediction of subsequent cardiovascular, and overall death compared with other indices of insulin resistance (Table 4). C‐peptide levels (AUC=0.62) had significantly higher AUC's compared with fasting plasma glucose levels (AUC=0.56), HOMA‐IR index (AUC=0.58), fasting serum insulin levels (AUC=0.57), QUICKI index (AUC=0.42), and metabolic syndrome (AUC=0.56) for cardiovascular death, with *P* values at <0.001. A similar pattern was found for overall mortality (AUC=0.60 for C‐peptide levels vs AUC=0.57 or less in the rest).

**Table 4.** Receiver Operating Characteristic Curves of Different Indices of Insulin Resistance to Predict Cardiovascular or Overall Death

Indices of Insulin Resistance	Area Under the Curve (95% Confidence Interval)	*P* Value vs C‐Peptide
Cardiovascular death		
Fasting serum C‐peptide levels, nmol/L	0.624 (0.624 to 0.625)	—
Fasting plasma glucose levels, mg/dL	0.558 (0.558 to 0.558)	<0.001
HOMA‐IR index	0.583 (0.583 to 0.583)	<0.001
Fasting serum insulin levels, pmol/L	0.567 (0.566 to 0.567)	<0.001
QUICKI index	0.417 (0.417 to 0.418)	<0.001
Metabolic syndrome	0.563 (0.563 to 0.563)	<0.001
Overall mortality		
Fasting serum C‐peptide levels, nmol/L	0.600 (0.600 to 0.600)	—
Fasting plasma glucose levels, mg/dL	0.566 (0.566 to 0.566)	<0.001
HOMA‐IR index	0.559 (0.558 to 0.559)	<0.001
Fasting serum insulin levels, pmol/L	0.549 (0.549 to 0.549)	<0.001
QUICKI index	0.442 (0.441 to 0.442)	<0.001
Metabolic syndrome	0.543 (0.543 to 0.543)	<0.001

Analysis was frequency weighted using NHANES 3 probability weights rounded to the nearest integer. Some 95% confidence intervals are extremely narrow or showed no variation due to rounding to the nearest 3 decimal points.

HOMA‐IR indicates homeostatic model assessment of insulin resistance; QUICKI, quantitative insulin sensitivity check index.

HOMA=fasting plasma glucose (mg/dL)×fasting serum insulin levels/405.

QUICKI index=1/(log [fasting serum insulin]+log [fasting plasma glucose]).

Metabolic syndrome by ATP3 criteria is diagnosed when ≥3 of the following criteria are present: waist circumference >102 cm for men and >88 cm for women, triglycerides level >150 mg/dL, high‐density lipoprotein level <40 mg/dL for men and <50 mg/dL for women, blood pressure ≥130/≥85 mm Hg, and fasting glucose ≥110 mg/dL.

### C‐Peptide Levels and Mortality

**Figure 1.**
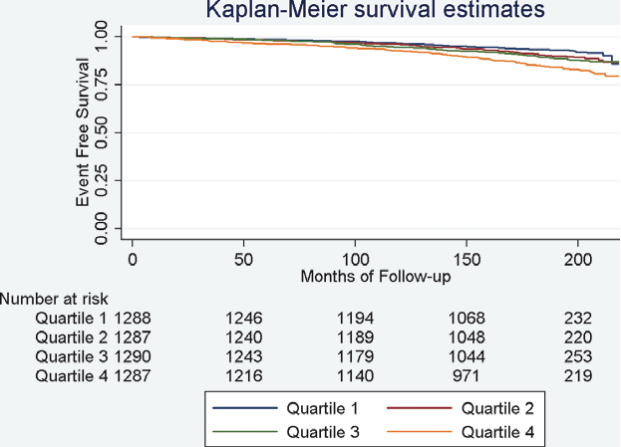
Kaplan–Meier Curve and percent survival estimates for cardiovascular disease death according to quartiles of C‐peptide levels. Quartile 1=0.418 nmol/L or less, Quartile 2=0.419 to 0.652 nmol/L, Quartile 3=0.653 to 0.983 nmol/L, Quartile 4=0.984 nmol/L or greater.

The median follow‐up for the study was 14.4 years (interquartile range: 3.3 years), with a total follow‐up of 70 768 person‐years. A total of 513 deaths occurred during the follow‐up period. Crude cumulative mortality rates increased significantly with increasing quartiles of C‐peptide levels for cardiovascular death (Figure 1), cerebrovascular disease death, ischemic heart disease death, myocardial infarction death, and overall mortality (*P* values <0.01 for all, Table 5). Of note, cardiovascular mortality was only one‐third of the overall mortality at the lowest C‐peptide quartile, but they were approximately half of the overall mortality for individuals at the highest C‐peptide quartile. The association between C‐peptide quartiles and mortality persisted even after adjustment for potential confounding factors (Table 5). When compared with individuals in the lowest quartile of C‐peptide levels (≤0.418 nmol/L), subjects in the highest C‐peptide quartile (≥0.984 nmol/L) had a 60% increase in the adjusted hazards of cardiovascular death. A similar pattern and magnitude of increased hazards of mortality for individuals in the highest C‐peptide quartile were found for cerebrovascular disease, ischemic heart disease, myocardial infarction, and overall mortality compared with participants in the lowest quartile of C‐peptide levels. Of note, analyses of specific outcomes such as death due to ischemic heart disease and myocardial infarction did not reach statistical significance, except for cerebrovascular disease death (adjusted HR=3.94, 95% confidence interval [CI] 1.28 to 12.1), although their 95% CIs of these specific outcomes overlap with those of cardiovascular and overall mortality (Table 5). The attributable risk percent for individuals with C‐peptide levels in the highest quartile vs the remaining of the population was 52% for cardiovascular mortality and 40.6% for overall mortality.

**Table 5.** HRs for Mortality Outcomes During the Study Period, According to Quartiles of C‐Peptide Levels

C‐Peptide Category, nmol/L					
	Overall	≤0.418	0.419 to 0.652	0.653 to 0.983	≥0.984
No. of participants	5140	1284	1283	1290	1283
Cardiovascular mortality, %*	7.2	3.8	6.0	7.9	12.2
Crude HR for cardiovascular mortality (95% CI)		Referent	**1.61 (1.13 to 2.33)**	**2.18 (1.40 to 3.28)**	**3.44 (2.38 to 5.03)**
Adjusted HR for cardiovascular mortality (95% CI)		Referent	1.20 (0.80 to 1.80)	1.42 (0.90 to 2.26)	**1.60 (1.07 to 2.39)**
No. of participants	5140	1284	1283	1290	1283
Cerebrovascular disease mortality, %*	1.0	0.5	0.6	0.8	2.2
Crude HR for cerebrovascular disease mortality (95% CI)		Referent	**1.33 (0.53 to 3.37)**	**1.83 (0.60 to 5.57)**	**4.82 (1.81 to 12.8)**
Adjusted HR for cerebrovascular disease mortality (95% CI)		Referent	1.12 (0.44 to 2.83)	1.22 (0.28 to 5.34)	**3.94 (1.28 to 12.1)**
No. of participants	5140	1284	1283	1290	1283
Ischemic heart disease mortality, %*	3.9	2.1	3.5	4.2	6.3
Crude HR for ischemic heart disease mortality (95% CI)		Referent	**1.67 (1.04 to 2.69)**	**2.05 (1.30 to 3.26)**	**3.15 (2.07 to 4.81)**
Adjusted HR for ischemic heart disease mortality (95% CI)		Referent	1.17 (0.66 to 2.10)	1.16 (0.65 to 2.06)	1.22 (0.67 to 2.21)
No. of participants	5140	1284	1283	1290	1283
Myocardial infarction mortality, %*	1.5	1.0	1.3	1.1	2.9
Crude HR for myocardial infarction mortality (95% CI)		Referent	**1.22 (0.59 to 2.51)**	**1.13 (0.44 to 2.92)**	**3.00 (1.32 to 6.79)**
Adjusted HR for myocardial infarction mortality (95% CI)		Referent	0.79 (0.37 to 1.67)	0.71 (0.25 to 2.01)	1.37 (0.66 to 2.83)
No. of participants	5152	1288	1287	1290	1287
Overall mortality, %*	18.1	12.3	14.7	20.6	26.4
Crude HR for overall mortality (95% CI)		Referent	1.22 (0.94 to 1.57)	**1.77 (1.42 to 2.18)**	**2.31 (1.77 to 2.99)**
Adjusted HR for overall mortality (95% CI)		Referent	1.12 (0.88 to 1.43)	**1.57 (1.27 to 1.95)**	**1.72 (1.34 to 2.21)**

ICD‐10 codes were used to define underlying cause of death. Cardiovascular mortality was defined as cardiovascular disease (I00 to I99) being the underlying cause of death or when it was one of the multiple causes of the death (American Heart Association 2011 update on heart disease and stroke).^16^ Cerebrovascular disease, ischemic heart disease, and myocardial infarction deaths are part of the cardiovascular disease death. Myocardial infarction death is part of the ischemic heart disease death.

Model was adjusted for age, sex, race, waist‐to‐hip ratio, body mass index, blood pressure, total cholesterol, triglycerides, high‐density lipoprotein, history of stroke, heart attack, peripheral arterial disease, family history of diabetes and heart attack, history of chest pain, level of education, smoking status, level of physical activity, alcohol use in last month, C‐reactive protein level, urinary albumin/creatinine ratio, glomerular filtration rate, and glycated hemoglobin levels. Bold values represent statistically significant results at *P* values of <0.05.

* *P* value <0.01 among quartiles. Mortality rates are population‐weighted averages calculated based on NHANES 3 probability weights rounded to the nearest integer.

Although additional analyses did not show a significant interaction between body mass index, sex, or race, and C‐peptide levels, we did find an interaction of borderline significance (*P*=0.04) between kidney function and the highest quartile of C‐peptide levels. Indeed, for subjects with a glomerular filtration rate <60 mL/min, the adjusted HR of cardiovascular death were 3.73 (95% CI 1.18 to 11.7) for individuals in the highest vs the lowest quartiles of C‐peptide levels. On the other hand, for subjects with a glomerular filtration rate ≥60 mL/min, the adjusted HR of cardiovascular death were nonsignificantly elevated at 1.34 (0.82 to 2.19) for individuals in the highest vs the lowest quartiles of C‐peptide levels. Subgroup analyses on individuals with a normal fasting glucose and in those without metabolic syndrome showed consistency of C‐peptide quartiles as an independent predictor of cardiovascular death (Table 6).

**Table 6.** Adjusted Hazard Ratio for Cardiovascular Death in Subgroups, According to Quartiles of C‐Peptide Levels

	Adjusted Hazard Ratios According Quartiles of C‐Peptide Levels, nmol/L (95% CI)			
	≤0.418	0.419 to 0.652	0.653 to 0.983	≥0.984
No metabolic syndrome* (n=3784)	Referent	1.32 (0.84 to 2.08)	**1.72 (1.10 to 2.69)**	**2.01 (1.29 to 3.11)**
Plasma glucose levels <100 mg/dL (n=3703)	Referent	1.20 (0.77 to 1.87)	1.34 (0.68 to 2.64)	**1.74 (1.02 to 2.95)**
Glomerular filtration rate ≥60 mL/min (n=3952)*	Referent	1.10 (0.68 to 1.80)	1.35 (0.84 to 2.16)	1.34 (0.82 to 2.19)
Glomerular filtration rate <60 mL/min (n=1115)*	Referent	2.63 (0.86 to 8.02)	3.03 (0.94 to 9.83)	**3.73 (1.18 to 11.7)**

Model was adjusted for age, sex, race, waist‐to‐hip ratio, body mass index, blood pressure (normal, prehypertension, hypertension), total cholesterol, triglycerides, high‐density lipoprotein, presence or absence of past history of stroke, heart attack, peripheral arterial disease, presence or absence of family history of diabetes and heart attack, history of chest pain (suggestive of angina, not suggestive of angina and no chest pain), level of education, smoking status, level of physical activity (no physical activity in last month, physical activity levels, alcohol use, C‐reactive protein level, urinary albumin/creatinine ratio, glomerular filtration rate, and glycated hemoglobin levels. Bold values represent statistically significant results at *P* values of <0.05.

* Metabolic syndrome is diagnosed based on ATP3 criteria, when ≥3 of the following criteria are present; waist circumference >102 cm for men and >88 cm for women, triglycerides level >150 mg/dL, high‐density lipoprotein level <40 mg/dL for men and <50 mg/dL for women, blood pressure ≥130/≥85 mm Hg, and fasting glucose ≥110 mg/dL (Third report of the National Cholesterol Education Program expert panel on detection, evaluation, and treatment of high blood cholesterol in adults).^5^

* Interaction glomerular filtration rate·fourth quartile of C‐peptide level was significant at a *P* value <0.04.

From the Cox model, independent clinical predictors associated with higher hazards of cardiovascular death were increasing age, male, blood pressure ≥140 mm Hg systolic or 90 mm Hg diastolic, education less than high school vs college level, current smoking, previous stroke, previous heart attack, and peripheral arterial disease. Independent biochemical predictors of cardiovascular and/or diabetes death included C‐reactive protein levels, urinary albumin/creatinine ratio, and C‐peptide levels (Table 7). Neither A1c, body mass index, nor fasting plasma glucose levels was an independent predictor of cardiovascular death in the model.

**Table 7.** Multivariate Adjusted HRs of Independent Covariate Predictors of Cardiovascular Death

Independent Predictors	Adjusted HRs (95% CI)
Age, y	1.10 (1.08 to 1.12)
Male	1.65 (1.23 to 2.21)
Blood pressure ≥140 mm Hg systolic or 90 mm Hg diastolic	2.00 (1.45 to 2.77)
Education less than high school versus college level	1.42 (1.04 to 1.95)
Current smoking	1.96 (1.33 to 2.88)
Previous stroke	2.32 (1.45 to 3.71)
Previous heart attack	2.51 (1.67 to 3.76)
Peripheral arterial disease	2.73 (1.32 to 5.64
C reactive protein levels, mg/dL	1.21 (1.03 to 1.42)
Urinary albumin/creatinine ratio	1.001 (1.001 to 1.001)

Model was adjusted for age, sex, race, waist‐to‐hip ratio, body mass index, blood pressure, total cholesterol, triglycerides, high‐density lipoprotein, history of stroke, heart attack, peripheral arterial disease, family history of diabetes or heart attack, history of chest pain, level of education, smoking status, level of physical activity, alcohol use in last month, C‐reactive protein level, urinary albumin/creatinine ratio, glomerular filtration rate, and glycated hemoglobin levels.

When other biochemical indices of insulin resistance such as serum insulin levels, HOMA‐IR, and QUICKI were used in lieu of quartiles C‐peptide levels in the study survival models, we only found a significant association between these other biomarkers and overall mortality but not with cardiovascular mortality (Table 8).

**Table 8.** HRs for Mortality Outcomes During the Study Period, According to Other Biochemical Indices of Insulin Resistance (N=5152)

	Fasting Insulin Levels, pmol/L	HOMA‐IR	QUICKI
Adjusted HR for cardiovascular mortality (95% CI)			
Quartile 1	Referent	Referent	Referent
Quartile 2	0.85 (0.59 to 1.21)	0.93 (0.61 to 1.42)	1.00 (0.71 to 1.40)
Quartile 3	1.10 (0.70 to 1.71)	1.13 (0.73 to 1.74)	0.82 (0.57 to 1.18)
Quartile 4	1.08 (0.66 to 1.77)	1.13 (0.72 to 1.78)	0.88 (0.56 to 1.38)
Adjusted HR for overall mortality (95% CI)			
Quartile 1	Referent	Referent	Referent
Quartile 2	0.95 (0.81 to 1.12)	1.00 (0.83 to 1.20)	0.95 (0.78 to 1.15)
Quartile 3	1.29 (0.98 to 1.71)	**1.29 (1.02** to **1.64)**	**0.74 (0.56** to **0.97)**
Quartile 4	**1.35 (1.00** to **1.81)**	**1.36 (1.01** to **1.82)**	**0.74 (0.55** to **0.99)**

CI indicates confidence interval; HR, hazard ratio; HOMA‐IR, fasting plasma glucose (mg/dL)×fasting serum insulin levels/405; QUICKI index, 1/[log (fasting serum insulin)+log (fasting plasma glucose)].

Model was adjusted for age, sex, race, waist‐to‐hip ratio, body mass index, blood pressure, total cholesterol, triglycerides, high density lipoprotein, history of stroke, heart attack, peripheral arterial disease, family history of diabetes and heart attack, history of chest pain, level of education, smoking status, level of physical activity, alcohol use in last month, C‐reactive protein level, urinary albumin/creatinine ratio, glomerular filtration rate, and glycated hemoglobin levels. Bold values represent statistically significant results at *P* values of <0.05.

## Discussion

Very few studies, if any, evaluated the role of fasting serum C‐peptide as a biomarker for future cardiovascular death. The present study suggests that fasting serum C‐peptide levels were a better predictor of cardiovascular and overall death than fasting serum insulin and its derived measures of insulin resistance in a nationwide sample of nondiabetic adults between 40 and 74 years of age. The effects were independent of several known risk factors for development of diabetes or cardiovascular disease and were consistent even in patients without metabolic syndrome or with normoglycemia. The increased risk of cardiovascular or overall death was 60% and 72% higher, respectively, for participants in the highest vs lowest C‐peptide quartiles, with consistent patterns observed in subgroup analyses for mortality due to cerebrovascular disease, ischemic heart disease, or myocardial infarction.

Substantial evidence supports the role of insulin resistance in the development of type 2 diabetes,^7,39^ but a relationship between insulin resistance‐hyperinsulinemia as measured by C‐peptide levels in adults without diabetes and a future cardiovascular death has not been established from our literature review. This association remained significant for adults without clinical manifestations of insulin resistance such as those with normal fasting glucose levels or without metabolic syndrome at the time of C‐peptide level testing. Thus, these results support the role of C‐peptide levels to be an important predictor of mortality. Studies in women with breast cancer have also used C‐peptide levels as an important prognostic indicator.^11,40^ Irwin et al^11^ use C‐peptide to track the metabolic profile of patients with breast cancer, because both glucose and lipid dysregulation have been associated with incidence of breast cancer.^41–42^ The authors found an increased risk of overall and breast cancer mortality associated with higher C‐peptide levels in the population of nondiabetic women with breast cancer.^11^ Our study builds on these results by showing that the increased mortality risk associated with high C‐peptide levels applied to both men and women of the general population between 40 and 74 years of age. Other indices of insulin resistance such as HOMA‐IR, that uses a product of serum insulin and plasma glucose levels, have also been shown to be an adverse prognostic indicator for cardiovascular and overall mortality.^10^ However, the utility of direct serum insulin measurements is limited by its short half‐life.^12–14^ In addition to supporting the increased mortality risk associated with an insulin resistant state with C‐peptide levels, we found C‐peptide levels to be a better predictor of cardiovascular and overall mortality compared with serum insulin levels. Indeed, C‐peptide levels outperformed other known markers of insulin resistance such as HOMA‐IR, QUICKI, and metabolic syndrome, among others, in the risk prediction models of cardiovascular and overall death. When compared with other independent predictors of cardiovascular death found in our study, the increased hazards associated with the highest vs the lowest C‐peptide quartile were comparable to that of being of male gender or a current smoker or to have elevated C‐reactive protein levels. Our findings suggest that the attributable risk of cardiovascular death associated with C‐peptide levels in the upper quartile of the population is ≈52%.

The association between high C‐peptide levels and increased risk of cardiovascular death likely reflects the association between insulin resistance and an atherogenic state.^43^ This hypothesis is supported by the majority of deaths associated with high C‐peptide levels being attributed to macrovascular disease (cerebrovascular and ischemic heart disease, and myocardial infarction) death. Although the results might appear that the point estimate for cerebrovascular disease death was higher than the ischemic heart disease or myocardial infarction death, the event rates were low and the 95% CIs overlap to avoid such conclusions. Future studies will be needed to further investigate the relationship between C‐peptide levels and specific macrovascular disease states. Other explanations for the increased risk of death associated with high C‐peptide levels despite adjustment for known cardiovascular risk factors may be that high C‐peptide levels predict the future worsening of the current risk factors and/or the future development of diabetes or cardiovascular disease.^7–8^ Another possibility is the existence of other significant predictors of cardiovascular death that we did not account for in our Cox model, such as diastolic dysfunction,^44^ endothelial dysfunction,^45^ and prothrombotic states.^46–47^

In addition, we found a weak but significant interaction between kidney function and high C‐peptide levels, where the cardiovascular mortality risk associated with high C‐peptide levels was significantly more accentuated among individuals with glomerular filtration rates <60 mL/min. Potential mechanisms for this interaction could be the increased endothelial dysfunction, inflammation and oxidative stress, among other cardiovascular risk factors that coexist in individuals with kidney disease,^48–50^ in addition to the excess cardiovascular risk associated with insulin resistance. On the other hand, our results did not show a significant interaction between C‐peptide levels and body mass index, which is in contrast with the report by Ausk et al,^10^ who found HOMA‐IR a significant predictor of overall mortality but only in individuals with a normal body mass index. While this discrepancy may be partly explained by the older age of our study population (40 to 74 years vs ≥20 years in the Ausk et al report), it is also possible that fasting serum C‐peptide level is a more stable measure of insulin secretion and, therefore, produced more robust results across different stages of obesity than HOMA‐IR.^9,13–14^

The results of this study should be interpreted in the context of several limitations. First, despite the significant relationship between C‐peptide levels and mortality, the power of discrimination of C‐peptide levels on the cardiovascular and overall mortality is only modest with AUCs of 0.62 and 0.60, respectively. Nevertheless, the ROC for C‐peptide was significantly better than all other indices of insulin resistance for the outcomes studied. Second, although we had data on deaths and the causes of death, we could not evaluate data on nonfatal cardiovascular events, which could have increased the power to detect smaller differences between exposure groups and shed light on the potential new mechanisms of harm from insulin resistance. Third, given the observational nature of our study, the possibility of residual confounding exists. Despite our efforts using both multivariate adjustment for several demographic and clinical known confounders for cardiovascular disease and diabetes, and stratified analyses to control for the influence of potential confounding, the possibility for “residual confounding” in our statistical modeling may still exist and should be taken into account in the interpretation of our results. Fourth, the use of death certificates to adjudicate causes of death may lead to potential misclassification.^51–53^ Despite this limitation, the accuracy of death certificates is higher for deaths related to diseases of the circulatory system, with a sensitivity of 82% and a positive predictive value of 75%.^53^ Finally, repetitive measurements of the different biological measures were not available. Ideally, the analysis of the cardiovascular death outcome should be repeated after exclusion of individuals who developed diabetes during follow‐up, which is one of the potential mechanisms at which insulin resistance could lead to cardiovascular death. Therefore, the current study could not discern whether the increased cardiovascular death risk in individuals with insulin resistance was due to the insulin resistance status per se or due to future development of risk factors such as diabetes. However, our analyses adjusted for several known risk factors for development of diabetes and subgroup analyses in individuals with lower risk of future diabetes, such as those without metabolic syndrome and those with normal fasting plasma glucose levels, consistently showed increased cardiovascular death risk among study participants in the highest quartile of C‐peptide levels. These results would support that the increased risk of cardiovascular death in subjects with high C‐peptide levels is unlikely to be attributed solely to the eventual development of diabetes, and suggest additional or alternative mechanisms of harm.

In summary, C‐peptide was superior to other insulin‐derived measures of insulin resistance in predicting cardiovascular and overall death in nondiabetic adults. C‐peptide predicted cardiovascular death even in subjects with normoglycemia and without metabolic syndrome. Prospective studies are needed to elucidate the usefulness of C‐peptide levels as a target for intervention to improve cardiovascular outcomes.
